# Supplementary material for: Application of Proteogenomics to Urine Analysis towards the Identification of Novel Biomarkers of Prostate Cancer: An Exploratory Study
Source: Cancers (Basel). 2022 Apr 15;14(8):2001. doi: 10.3390/cancers14082001 (PMC9031064; doi:10.3390/cancers14082001)
Supplement: Supplementary file 1 [file cancers-14-02001-s001.zip › cancers-1629804-supplementary.pdf]

Table S1. Clinical data of subjects included in the discovery cohort group.

**Characteristic**	**PCa, *n* = 5**	**Controls, *n* = 5**
Age years	60 ± 2.5	59 ± 5.4
Serum PSA_ ng/mL	18 ± 14	-
Combined Gleason Score	7 (4 + 3): 2 (40%)<br>7 (3 + 4): 1 (20%)<br>6 (3 + 3): 2 (40%)	-
Clinical Stage	cT2a: 2 (40%)<br>cT2b: 3 (60%)	-
Other pathologies		
Yes	AH+ Dyslipidemia: 1 (20%)<br>AH+ Diabetes: 1 (20%)<br>Hyperuricemia: 1 (20%)	AH: 2 (40%)
No	2 (40%)	3 (60%)
Smoking habits		
Yes	1 (20%)	3 (60%)
No	1 (20%)	2 (40%)
Unknown	3 (60%)	-

Based on a shotgun discovery experiment, some candidate urinary targets for PCa prediction were identified in the discovery cohort (composed of five PCa patients (PCa 1-5d) and five non-cancer subjects (CNT 1-5d)). CT2a patients present a tumour in one half or less of only one side of the prostate, while, in CT2b stage, the tumour is localized in more than half of one side of the prostate. On the other hand, in CT3a and CT3b patients, the tumour extends beyond the prostate capsule or invades seminal vesicle(s), respectively. Abbreviations: AH: Arterial hypertension; CNT, cancer-free subjects; PCa, prostate cancer patients. .

Table S2. Clinical data of subjects included in the testing cohort group.

**Characteristic**	**PCa, *n* = 30**	**Controls, *n* = 30**
Age_years	62 ± 8.8	58 ± 3.3
Serum PSA_ ng/mL	12 ± 11	-
Combined Gleason Score		
Known	6 (3 + 3): 7 (23.33%)<br>7 (3 + 4): 11 (36.67%)<br>7 (4 + 3): 3 (10%)<br>8 (4 + 4): 4 (13.33%)<br>9 (4 + 5): 3 (10%)<br>9 (5 + 4): 1 (3.33%)	-
Unknown	1 (3.33%)	-
Clinical Stage		-
Known	cT1N0M0: 2 (6.67%)<br>cT2N0M0: 13 (43.33%)<br>cT2bN0M0: 2 (6.67%)<br>cT3N1M0: 2 (6.67%)<br>cT3aN0M0: 4 (13.33%)<br>cT3bN0M0: 3 (10%)<br>cT3bN1M0: 1 (3.33%)<br>cT3bN + M0: 1 (3.33%)	-
Unknown	2 (6.67%)	-
Other pathologies		

Characteristic	PCa, *n* = 30	Controls, *n* = 30
Yes	Dyslipidemia: 4 (13.33%)<br>AH: 2 (6.67%)<br>T2D: 1 (3.33%)<br>AH+ Dyslipidemia: 3 (10%)<br>T2D+Dyslipidemia+AH: 1 (3.33%)<br>AH+ Hyperuricemia: 1 (3.33%)<br>AH+ Hyperuricemia+ Dyslipidemia: 1 (3.33%)<br>T2D + Dyslipidemia + Hyperuricemia: 1 (3.33%)<br>T2D; Dyslipidemia: 1 (3.33%)	-
Unknown	15 (50%)	30 (100%)
Smoking habits		
Yes	10 (33.33%)	13 (43.33%)
No	13 (43.33%)	17 (56.67%)
Unknown	7 (23.33%)	-
BMI	26 ± 2.3	-

The suspected biomarkers were evaluated in an independent testing group, comprising thirty PCa patients (PCa 1-30) and thirty cancer-free subjects (CNT1-30). CT2a patients present a tumour in one half or less of only one side of the prostate, while, in CT2b stage, the tumour is localized in more than half of one side of the prostate. On the other hand, in CT3a and CT3b patients, the tumour extends beyond the prostate capsule or invades seminal vesicle(s), respectively. Concerning regional lymph nodes: Nx, defines a tumour that cannot be measured in nearby lymph nodes; N0, a tumour that has not spread to regional lymph nodes and N1, a tumour that has spread to regional lymph nodes. M1 and M0 nomenclature represent the tumours that have not spread to other parts of the body, respectively. Abbreviations: AH: Arterial hypertension; BMI: Body mass index; CNT, cancer-free subjects; PCa, prostate cancer patients; T2D: Type 2 diabetes. .

Table S3. Summary of statistical analysis results of shortlisted proteins evaluated in the testing group.

Characteristic	N	Overall, *n* = 60 [1]	PCa, *n* = 30 [1]	Controls, *n* = 30 [1]	*p*-value [2]
Age_years	60	59 (56, 65)	60 (57, 70)	58(56, 60)	0.04
Smoking_habits	60				
Unknown		7 (12%)	7 (23.33%)	0 (0%)	
No		30 (50%)	13 (43.33%)	17 (57%)	
Yes		23 (38%)	10 (33.33%)	13 (43%)	
AMBP	60	0.95 (0.79, 1.13)	0.99 (0.81, 1.14)	0.94 (0.80, 1.12)	0.65
CDH1	60	0.93 (0.59, 1.60)	0.42 (0.31, 0.56)	0.43 (0.36, 0.57)	0.75
EFEMP1	60	0.43 (0.33, 0.56)	0.42 (0.31, 0.56)	0.43 (0.36, 0.57)	0.46
LMAN2	60	0.75 (0.65, 0.96)	0.76 (0.71, 1.02)	0.70 (0.62, 0.87)	0.17
TTR	60	0.47 (0.28, 0.84)	0.49 (0.28, 0.80)	0.40 (0.30, 0.88)	0.84

1. Median (IQ1, IQ3). **2**. Wilcoxon rank sum test; t-test with Welch´s correction; Student's t-Test.

Table S5. Prediction of likely impact of point mutations on protein function using PolyPhen-2 tool.

Protein name	Gene name	Mutation	Prediction	Score	Sensitivity	Specificity
Nidogen-1	NID1	P99T	Possibly damaging	0.722	0.86	0.92
Nidogen-1	NID1	V246I	Benign	0	1	0
Lactotransferrin	LTF	A29T	Benign	0.001	0.99	0.15
Lactotransferrin	LTF	E579D	Benign	0.001	0.99	0.15
Lactotransferrin	LTF	P444T	Benign	0.01	0.96	0.77
Centrosome-associated protein 350	CEP350	L2405F	Probably damaging	0.989	0.72	0.97

CLIP-associating protein 2	CLASP2	I201V	Probably damaging	0.989	0.72	0.97
Multivesicular body subunit 12B	MVB12B	T205M	Probably damaging	0.995	0.68	0.97
Prothrombin	F2	E110K	Benign	0.001	0.99	0.15
Zinc finger CCCH-type antiviral protein 1	ZC3HAV1	P209L	Benign	0.022	0.95	0.8
Tetranectin	CLEC3B	G106S	Benign	0.099	0.93	0.85
Alpha-2-HS-glycoprotein	AHSG	G75A	Possibly damaging	0.945	0.8	0.95
Aminopeptidase N	ANPEP	D370N	Possibly damaging	0.74	0.85	0.92
Aminopeptidase N	ANPEP	R86Q	Benign	0.001	0.99	0.15
Aminopeptidase N	ANPEP	P465L	Probably damaging	0.996	0.55	0.98
Aminopeptidase N	ANPEP	G141D	Possibly damaging	0.851	0.83	0.93
Deoxyribonuclease-1	DNASE1	G262D	Possibly damaging	0.489	0.89	0.9
tRNA pseudouridine(38/39) synthase	PUS3	Q126H	Probably damaging	1	0	1
Collagen alpha-1(VI) chain	COL6A1	G700V	Probably damaging	0.999	0.14	0.99
Alpha-1-antichymotrypsin	SERPINA3	E164Q	Probably damaging	0.996	0.55	0.98
Complement C3	C3	P314L	Benign	0.227	0.91	0.88
Complement C1r subcomponent-like protein	C1RL	I285V	Benign	0.45	0.89	0.9
Probable protein phosphatase 1N	PPM1N	T217A	Benign	0.27	0.91	0.88
Fibrinogen alpha chain	FGA	D633E	Benign	0.127	0.93	0.86
Iron-sulfur cluster assembly 2 homolog, mitochondrial	ISCA2	V121L	Possibly damaging	0.649	0.87	0.91
Sorting nexin-2	SNX2	Q312K	Possibly damaging	0.578	0.88	0.91
Ephrin type-A receptor 7	EPHA7	E240K	Possibly damaging	0.545	0.88	0.91
Hemopexin	HPX	Y198C	Probably damaging	1	0	1
Hemopexin	HPX	P140R	Probably damaging	1	0	1
Acid-sensing ion channel 3	ASIC3	S156F	Possibly damaging	0.831	0.84	0.93
Plasma serine protease inhibitor	SERPINA5	S68F	Probably damaging	1	0	1
Plasma serine protease inhibitor	SERPINA5	A60D	Benign	0.002	0.99	0.3
Plasma serine protease inhibitor	SERPINA5	K105E	Benign	0	1	0

Alpha-1-acid glycoprotein 2	ORM2	S143Y	Probably damaging	0.984	0.74	0.96
Endosialin	CD248	S72N	Benign	0	1	0
Endosialin	CD248	A303V	Probably damaging	0.957	0.78	0.95
Lysosomal alpha-glucosidase	GAA	R223H	Benign	0.206	0.92	0.88
Lysosomal alpha-glucosidase	GAA	E689K	Benign	0.028	0.95	0.81
Lysosomal alpha-glucosidase	GAA	V780I	Benign	0.001	0.99	0.15
Lysosomal alpha-glucosidase	GAA	V84I	Benign	0.001	0.99	0.15
Keratin, type I cytoskeletal 17	KRT17	G231A	Benign	0.389	0.9	0.89
Keratin, type I cytoskeletal 17	KRT17	I208T	Possibly damaging	0.956	0.79	0.95
Prolactin-inducible protein	PIP	Q79H	Benign	0.001	0.99	0.15
NKAP-like protein	NKAPL	R6L	Possibly damaging	0.649	0.87	0.91
Ganglioside GM2 activator	GM2A	V153A	Benign	0.019	0.95	0.8
Catechol O-methyltransferase	COMT	A146V	Probably damaging	0.968	0.77	0.95
Glioma pathogenesis-related protein 1	GLIPR1	P223A	Possibly damaging	0.915	0.81	0.94
Zymogen granule protein 16 homolog B	ZG16B	D71N	Possibly damaging	0.899	0.82	0.94
Alpha-1-antitrypsin	SERPINA1	E400D	Benign	0.002	0.99	0.3
Elongator complex protein 4	ELP4	A405V	Possibly damaging	0.565	0.88	0.91
Polymeric immunoglobulin receptor	PIGR	G336D	Benign	0.092	0.93	0.85
Polymeric immunoglobulin receptor	PIGR	G365S	Probably damaging	1	0	1
Insulin-like growth factor-binding protein 7	IGFBP7	G244E	Probably damaging	1	0	1
Kininogen-1	KNG1	P154L	Probably damaging	0.998	0.21	0.99
Epididymis-specific alpha-mannosidase	MAN2B2	G624R	Probably damaging	0.998	0.27	0.99
Haptoglobin-related protein	HPR	V325M	Probably damaging	0.987	0.73	0.96
Colagem alpha-3(IV) chain	COL4A3	P1255T	Benign	0.267	0.91	0.88
Actin, cytoplasmic 2	ACTG1	G168A	Probably damaging	0.998	0.27	0.99
Actin, cytoplasmic 2	ACTG1	G156V	Probably damaging	1	0	1
Gamma-glutamyl hydrolase	GGH	L183R	Benign	0.001	0.99	0.15
Leucine-rich alpha-2-glycoprotein	LRG1	P133S	Benign	0.267	0.91	0.88

Beta-hexosaminidase subunit alpha	HEXA	I436V	Benign	0	1	0
Retinol-binding protein 4	RBP4	G93D	Probably damaging	1	0	1
Fibronectin	FN1	V2261I	Benign	0.002	0.99	0.3
Glutaminyl-peptide cyclotransferase	QPCT	L320R	Probably damaging	0.998	0.27	0.99
BDNF/NT-3 growth factors receptor	NTRK2	K328R	Possibly damaging	0.605	0.87	0.91
Vitamin D-binding protein	GC	D432E	Benign	0	1	0
CUB and sushi domain-containing protein 2	CSMD2	L3294F	Possibly damaging	0.533	0.88	0.9
Argininosuccinate synthase	ASS1	E256K	Benign	0.004	0.97	0.59
Protein FAM110D	FAM110	P153S	Possibly damaging	0.476	0.89	0.9
Dipeptidyl peptidase 1	CTSC	I153T	Benign	0	1	0
Septin-7	SEPTIN7	R147L	Probably damaging	0.995	0.68	0.97
Myeloid cell surface antigen CD33	CD33	R69G	Benign	0.016	0.95	0.79
CMRF35-like molecule 9	CD300LG	R60P	Benign	0.14	0.92	0.86
Plectin	PLEC	A641V	Benign	0	1	0
Basal cell adhesion molecule	BCAM	G85D	Probably damaging	0.998	0.27	0.99
Acetyl-CoA carboxylase 2	ACACB	R1298Q	Probably damaging	1	0	1
Tropomyosin alpha-3 chain	TPM3	R245G				
Nuclear mitotic apparatus protein 1	NUMA1	E1479Q	Benign	0.4	0.9	0.9
Collagen alpha-1(V) chain	COL5A1	E947K	Possibly damaging	0.956	0.79	0.95
C-type lectin domain family 14 member A	CLEC14A	D337G	Benign	0	1	0

Table S7. Prediction of likely impact of point mutations on protein-protein interactions using SAAMBE-SEQ tool.

Protein (Gene name) 1	Protein (Gene name) 2	Prediction
Protein AMBP (AMBP)	Lactotransferrin (LTF)	Position Wild Mutant ddG Type<br>69 V M 0.47 Destabilizing<br>192 E G 0.72 Destabilizing<br>**286 A G 0.56 Destabilizing**<br>238 G S 0.58 Destabilizing<br>197 P S 0.86 Destabilizing<br>338 G S 0.65 Destabilizing<br>341 G A 0.70 Destabilizing<br>313 V I 1.29 Destabilizing<br>186 G R -0.36 Stabilizing<br>185 R Q 1.31 Destabilizing
Protein AMBP (AMBP)	Complement C3 (C3)	Position Wild Mutant ddG Type<br>69 V M 0.23 Destabilizing<br>192 E G 0.56 Destabilizing<br>**286 A G 0.44 Destabilizing**

		238 G S 0.36 Destabilizing<br>197 P S 0.78 Destabilizing<br>338 G S 0.68 Destabilizing<br>341 G A 0.69 Destabilizing<br>313 V I 1.18 Destabilizing<br>186 G R -0.27 Stabilizing<br>185 R Q 1.19 Destabilizing
Protein AMBP (AMBP)	Myeloid cell surface antigen CD33 (CD33)	Position Wild Mutant ddG Type<br>69 V M 0.31 Destabilizing<br>192 E G 0.68 Destabilizing<br>**286 A G 0.64 Destabilizing**<br>238 G S 0.30 Destabilizing<br>197 P S 0.79 Destabilizing<br>338 G S 0.55 Destabilizing<br>341 G A 0.79 Destabilizing<br>313 V I 0.88 Destabilizing<br>186 G R -0.17 Stabilizing<br>185 R Q 1.09 Destabilizing
Protein AMBP (AMBP)	Kininogen-1 (KNG1)	Position Wild Mutant ddG Type<br>69 V M 0.30 Destabilizing<br>192 E G 0.67 Destabilizing<br>**286 A G 0.37 Destabilizing**<br>238 G S 0.48 Destabilizing<br>197 P S 0.84 Destabilizing<br>338 G S 0.56 Destabilizing<br>341 G A 0.68 Destabilizing<br>313 V I 0.96 Destabilizing<br>186 G R -0.24 Stabilizing<br>185 R Q 1.31 Destabilizing
Protein AMBP (AMBP)	Alpha-1-antichymotrypsin (SERPINA3)	Position Wild Mutant ddG Type<br>69 V M 0.43 Destabilizing<br>192 E G 0.68 Destabilizing<br>**286 A G 0.58 Destabilizing**<br>238 G S 0.29 Destabilizing<br>197 P S 0.77 Destabilizing<br>338 G S 0.96 Destabilizing<br>341 G A 0.91 Destabilizing<br>313 V I 0.94 Destabilizing<br>186 G R -0.03 Stabilizing<br>185 R Q 1.01 Destabilizing
Protein AMBP (AMBP)	Haptoglobin-related protein (HPR)	Position Wild Mutant ddG Type<br>69 V M 0.49 Destabilizing<br>192 E G 0.94 Destabilizing<br>**286 A G 0.80 Destabilizing**<br>238 G S 0.47 Destabilizing<br>197 P S 0.73 Destabilizing<br>338 G S 0.92 Destabilizing<br>341 G A 0.99 Destabilizing<br>313 V I 0.89 Destabilizing<br>186 G R -0.24 Stabilizing<br>185 R Q 1.27 Destabilizing

Protein AMBP (AMBP)	Osteopontin (SPP1)	Position Wild Mutant ddG Type<br>69 V M 0.18 Destabilizing<br>192 E G 0.77 Destabilizing<br>**286 A G 0.33 Destabilizing**<br>238 G S 0.37 Destabilizing<br>197 P S 0.75 Destabilizing<br>338 G S 0.39 Destabilizing<br>341 G A 0.56 Destabilizing<br>313 V I 0.89 Destabilizing<br>186 G R -0.22 Stabilizing<br>185 R Q 1.41 Destabilizing
Protein AMBP (AMBP)	Complement C1r subcomponent-like protein (C1RL)	Position Wild Mutant ddG Type<br>69 V M 0.39 Destabilizing<br>192 E G 0.86 Destabilizing<br>**286 A G 0.85 Destabilizing**<br>238 G S 0.45 Destabilizing<br>197 P S 0.81 Destabilizing<br>338 G S 0.80 Destabilizing<br>341 G A 0.93 Destabilizing<br>313 V I 1.01 Destabilizing<br>186 G R -0.24 Stabilizing<br>185 R Q 1.32 Destabilizing
Protein AMBP (AMBP)	Fibronectin (FN1)	Position Wild Mutant ddG Type<br>69 V M 0.32 Destabilizing<br>192 E G 0.80 Destabilizing<br>**286 A G 0.54 Destabilizing**<br>238 G S 0.59 Destabilizing<br>197 P S 0.72 Destabilizing<br>338 G S 0.93 Destabilizing<br>341 G A 0.94 Destabilizing<br>313 V I 1.13 Destabilizing<br>186 G R -0.23 Stabilizing<br>185 R Q 1.17 Destabilizing
Protein AMBP (AMBP)	Zinc-alpha-2-glycoprotein (AZGP1)	Position Wild Mutant ddG Type<br>69 V M 0.46 Destabilizing<br>192 E G 0.87 Destabilizing<br>**286 A G 0.56 Destabilizing**<br>238 G S 0.29 Destabilizing<br>197 P S 0.68 Destabilizing<br>338 G S 0.91 Destabilizing<br>341 G A 0.96 Destabilizing<br>313 V I 0.75 Destabilizing<br>186 G R 0.17 Destabilizing<br>185 R Q 0.90 Destabilizing
Protein AMBP (AMBP)	Retinol-binding protein 4 (RBP4)	Position Wild Mutant ddG Type<br>69 V M 0.47 Destabilizing<br>192 E G 0.90 Destabilizing<br>**286 A G 0.82 Destabilizing**<br>238 G S 0.59 Destabilizing<br>197 P S 1.00 Destabilizing<br>338 G S 0.83 Destabilizing<br>341 G A 0.96 Destabilizing

		313 V I 1.33 Destabilizing<br>186 G R -0.08 Stabilizing<br>185 R Q 1.42 Destabilizing
Protein AMBP (AMBP)	Hemopexin (HPX)	Position Wild Mutant ddG Type<br>69 V M 0.19 Destabilizing<br>192 E G 0.90 Destabilizing<br>**286 A G 0.64 Destabilizing**<br>238 G S 0.33 Destabilizing<br>197 P S 0.74 Destabilizing<br>338 G S 0.49 Destabilizing<br>341 G A 0.89 Destabilizing<br>313 V I 0.79 Destabilizing<br>186 G R -0.34 Stabilizing<br>185 R Q 1.31 Destabilizing
Protein AMBP (AMBP)	Plasma serine protease inhibitor (SERPINA5)	Position Wild Mutant ddG Type<br>69 V M 0.53 Destabilizing<br>192 E G 0.73 Destabilizing<br>**286 A G 0.66 Destabilizing**<br>238 G S 0.30 Destabilizing<br>197 P S 0.71 Destabilizing<br>338 G S 0.90 Destabilizing<br>341 G A 0.87 Destabilizing<br>313 V I 0.87 Destabilizing<br>186 G R -0.00 Stabilizing<br>185 R Q 1.06 Destabilizing
Protein AMBP (AMBP)	Alpha-1-antitrypsin (SERPINA1)	Position Wild Mutant ddG Type<br>69 V M 0.58 Destabilizing<br>192 E G 0.69 Destabilizing<br>**286 A G 0.62 Destabilizing**<br>238 G S 0.35 Destabilizing<br>197 P S 0.73 Destabilizing<br>338 G S 0.96 Destabilizing<br>341 G A 0.81 Destabilizing<br>313 V I 0.84 Destabilizing<br>186 G R 0.00 Destabilizing<br>185 R Q 1.02 Destabilizing
Protein AMBP (AMBP)	Vitamin D-binding protein (GC)	Position Wild Mutant ddG Type<br>69 V M 0.27 Destabilizing<br>192 E G 0.55 Destabilizing<br>**286 A G 0.28 Destabilizing**<br>238 G S 0.50 Destabilizing<br>197 P S 0.90 Destabilizing<br>338 G S 0.60 Destabilizing<br>341 G A 0.63 Destabilizing<br>313 V I 1.25 Destabilizing<br>186 G R -0.38 Stabilizing<br>185 R Q 1.12 Destabilizing
Protein AMBP (AMBP)	Alpha-1-acid glycoprotein 2 (ORM2)	Position Wild Mutant ddG Type<br>69 V M 0.26 Destabilizing<br>192 E G 0.52 Destabilizing<br>**286 A G 0.60 Destabilizing**<br>238 G S 0.44 Destabilizing

		197 P S 0.76 Destabilizing<br>338 G S 0.82 Destabilizing<br>341 G A 0.81 Destabilizing<br>313 V I 1.14 Destabilizing<br>186 G R -0.23 Stabilizing<br>185 R Q 0.99 Destabilizing
Protein AMBP (AMBP)	Inter-alpha-trypsin inhibitor heavy chain H4 (ITIH4)	Position Wild Mutant ddG Type<br>69 V M 0.57 Destabilizing<br>192 E G 0.96 Destabilizing<br>**286 A G 0.70 Destabilizing**<br>238 G S 0.63 Destabilizing<br>197 P S 1.11 Destabilizing<br>338 G S 0.91 Destabilizing<br>341 G A 0.95 Destabilizing<br>313 V I 1.53 Destabilizing<br>186 G R -0.02 Stabilizing<br>185 R Q 1.51 Destabilizing
Protein AMBP (AMBP)	Fibrinogen alpha chain (FGA)	Position Wild Mutant ddG Type<br>69 V M 0.40 Destabilizing<br>192 E G 0.81 Destabilizing<br>**286 A G 0.75 Destabilizing**<br>238 G S 0.36 Destabilizing<br>197 P S 0.90 Destabilizing<br>338 G S 0.72 Destabilizing<br>341 G A 0.92 Destabilizing<br>313 V I 1.19 Destabilizing<br>186 G R -0.04 Stabilizing<br>185 R Q 1.29 Destabilizing
Protein AMBP (AMBP)	Prothrombin (F2)	Position Wild Mutant ddG Type<br>69 V M 0.15 Destabilizing<br>192 E G 0.79 Destabilizing<br>**286 A G 0.61 Destabilizing**<br>238 G S 0.26 Destabilizing<br>197 P S 0.80 Destabilizing<br>338 G S 0.69 Destabilizing<br>341 G A 0.88 Destabilizing<br>313 V I 1.00 Destabilizing<br>186 G R -0.21 Stabilizing<br>185 R Q 1.23 Destabilizing
Basement membrane-specific heparan sulfate proteoglycan core protein (HSPG2)	Collagen alpha-3(IV) chain (COL4A3)	Position Wild Mutant ddG Type<br>**1062 Q H 0.70 Destabilizing**<br>4332 V I 1.08 Destabilizing<br>1503 A V 0.24 Destabilizing<br>970 S F 0.48 Destabilizing<br>638 M V 0.74 Destabilizing
Basement membrane-specific heparan sulfate proteoglycan core protein (HSPG2)	Collagen alpha-1(V) chain (COL5A1)	Position Wild Mutant ddG Type<br>**1062 Q H 0.66 Destabilizing**<br>4332 V I 1.18 Destabilizing<br>1503 A V 0.25 Destabilizing<br>970 S F 0.63 Destabilizing<br>638 M V 0.80 Destabilizing

Basement membrane-specific heparan sulfate proteoglycan core protein (HSPG2)	Collagen alpha-1(VI) chain (COL6A1)	Position Wild Mutant ddG Type<br>**1062 Q H 0.39 Destabilizing**<br>4332 V I 1.23 Destabilizing<br>1503 A V -0.42 Stabilizing<br>970 S F 0.56 Destabilizing<br>638 M V 0.74 Destabilizing
Basement membrane-specific heparan sulfate proteoglycan core protein (HSPG2)	Fibronectin (FN1)	Position Wild Mutant ddG Type<br>**1062 Q H 1.18 Destabilizing**<br>4332 V I 1.32 Destabilizing<br>1503 A V 0.15 Destabilizing<br>970 S F 0.70 Destabilizing<br>638 M V 1.01 Destabilizing
Basement membrane-specific heparan sulfate proteoglycan core protein (HSPG2)	Nidogen-1 (NID1)	Position Wild Mutant ddG Type<br>**1062 Q H 1.12 Destabilizing**<br>4332 V I 1.10 Destabilizing<br>1503 A V -0.14 Stabilizing<br>970 S F 0.52 Destabilizing<br>638 M V 0.94 Destabilizing
Basement membrane-specific heparan sulfate proteoglycan core protein (HSPG2)	Inter-alpha-trypsin inhibitor heavy chain H4 (ITIH4)	Position Wild Mutant ddG Type<br>**1062 Q H 0.88 Destabilizing**<br>4332 V I 1.48 Destabilizing<br>1503 A V 0.06 Destabilizing<br>970 S F 0.53 Destabilizing<br>638 M V 1.03 Destabilizing
Complement decay-accelerating factor (CD55)	Complement C3 (C3)	Position Wild Mutant ddG Type<br>**162 S L 0.74 Destabilizing**
Vasorin (VASN)	Fibronectin (FN1)	Position Wild Mutant ddG Type<br>**161 R Q 1.56 Destabilizing**
Fibronectin (FN1)	Insulin-like growth factor-binding protein 7 (IGFBP7)	Position Wild Mutant ddG Type<br>2261 V I 1.78 Destabilizing
Alpha-2-HS-glycoprotein (AHSG)	Osteopontin (SPP1)	Position Wild Mutant ddG Type<br>75 G A 0.83 Destabilizing
Fibronectin (FN1)	Prostate-specific antigen (KLK3)	Position Wild Mutant ddG Type<br>2261 V I 1.83 Destabilizing
Fibronectin (FN1)	Alpha-1-antitrypsin (SERPINA1)	Position Wild Mutant ddG Type<br>2261 V I 1.01 Destabilizing
Fibronectin (FN1)	Osteopontin (SPP1)	Position Wild Mutant ddG Type<br>2261 V I 1.36 Destabilizing
Alpha-2-HS-glycoprotein (AHSG)	Fibronectin (FN1)	Position Wild Mutant ddG Type<br>75 G A 1.36 Destabilizing
Complement C3 (C3)	Fibronectin (FN1)	Position Wild Mutant ddG Type<br>314 P L 0.38 Destabilizing
Prothrombin (F2)	Fibronectin (FN1)	Position Wild Mutant ddG Type<br>110 E K 1.11 Destabilizing
Fibrinogen alpha chain (FGA)	Fibronectin (FN1)	Position Wild Mutant ddG Type<br>633 D E 0.93 Destabilizing
Fibrinogen alpha chain (FGA)	Alpha-1-antitrypsin (SERPINA1)	Position Wild Mutant ddG Type<br>633 D E 0.12 Destabilizing
Alpha-2-HS-glycoprotein (AHSG)	Fibrinogen alpha chain (FGA)	Position Wild Mutant ddG Type<br>75 G A 1.03 Destabilizing
Complement C3 (C3)	Fibrinogen alpha chain (FGA)	Position Wild Mutant ddG Type<br>314 P L 0.42 Destabilizing

Prothrombin (F2)	Fibrinogen alpha chain (FGA)	Position Wild Mutant ddG Type<br>110 E K 0.93 Destabilizing
Prothrombin (F2)	Osteopontin (SPP1)	Position Wild Mutant ddG Type<br>110 E K 0.83 Destabilizing
Alpha-2-HS-glycoprotein (AHSG)	Prothrombin (F2)	Position Wild Mutant ddG Type<br>75 G A 1.06 Destabilizing
Complement C3 (C3)	Prothrombin (F2)	Position Wild Mutant ddG Type<br>314 P L 0.34 Destabilizing
Alpha-2-HS-glycoprotein (AHSG)	Complement C3 (C3)	Position Wild Mutant ddG Type<br>75 G A 0.84 Destabilizing
Alpha-2-HS-glycoprotein (AHSG)	Alpha-1-antitrypsin (SERPINA1)	Position Wild Mutant ddG Type<br>75 G A 1.01 Destabilizing
Complement C3 (C3)	Alpha-1-antitrypsin (SERPINA1)	Position Wild Mutant ddG Type<br>314 P L 0.25 Destabilizing
Prothrombin (F2)	Alpha-1-antitrypsin (SERPINA1)	Position Wild Mutant ddG Type<br>110 E K 0.76 Destabilizing
Prostate-specific antigen (KLK3)	Alpha-1-antitrypsin (SERPINA1)	Position Wild Mutant ddG Type<br>209 C Y 0.14 Destabilizing<br>156 G V 1.17 Destabilizing<br>55 V M 0.51 Destabilizing<br>117 S P 0.11 Destabilizing<br>87 G R 1.26 Destabilizing<br>124 L F 1.07 Destabilizing<br>154 A T 0.67 Destabilizing<br>179 I T 1.59 Destabilizing
Kininogen-1 (KNG1)	Alpha-1-antitrypsin (SERPINA1)	Position Wild Mutant ddG Type<br>154 P L 0.57 Destabilizing
Alpha-2-HS-glycoprotein (AHSG)	Kininogen-1 (KNG1)	Position Wild Mutant ddG Type<br>75 G A 0.83 Destabilizing
Complement C3 (C3)	Kininogen-1 (KNG1)	Position Wild Mutant ddG Type<br>314 P L 0.38 Destabilizing
Prothrombin (F2)	Kininogen-1 (KNG1)	Position Wild Mutant ddG Type<br>110 E K 0.77 Destabilizing
Fibrinogen alpha chain (FGA)	Kininogen-1 (KNG1)	Position Wild Mutant ddG Type<br>633 D E 0.44 Destabilizing
Fibronectin (FN1)	Kininogen-1 (KNG1)	Position Wild Mutant ddG Type<br>2261 V I 1.45 Destabilizing
Prostate-specific antigen (KLK3)	Kininogen-1 (KNG1)	Position Wild Mutant ddG Type<br>209 C Y 0.52 Destabilizing<br>156 G V 0.90 Destabilizing<br>55 V M 0.66 Destabilizing<br>117 S P 0.16 Destabilizing<br>87 G R 1.06 Destabilizing<br>124 L F 0.79 Destabilizing<br>154 A T 0.48 Destabilizing<br>179 I T 1.48 Destabilizing

Protein AMBP (AMBP)

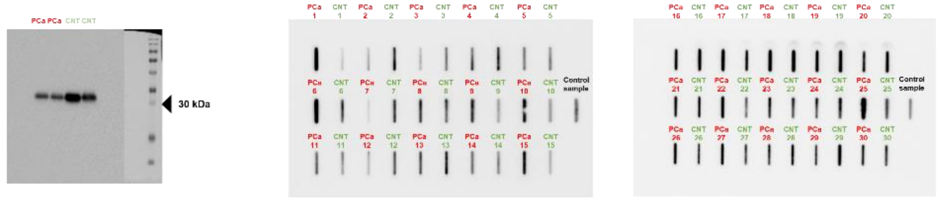

Vesicular integral-membrane protein VIP36 (LMAN2)

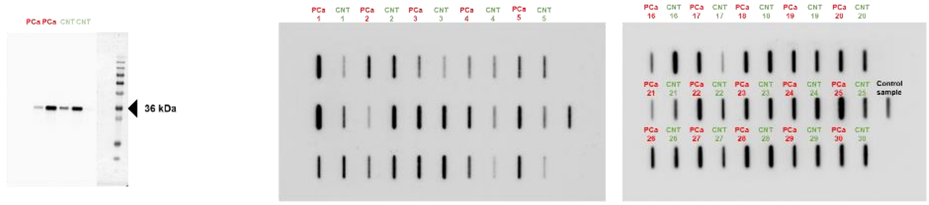

EGF-containing fibulin-like extracellular matrix protein 1 (EFEMP1)

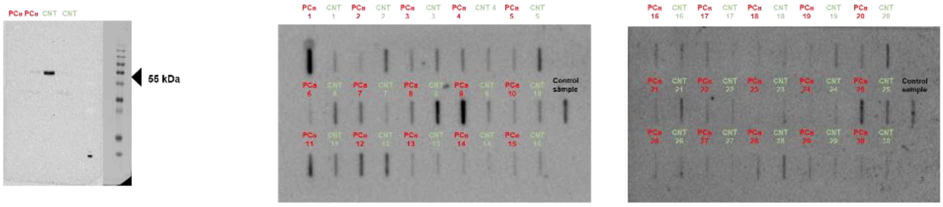

Cadherin-1 (CDH1)

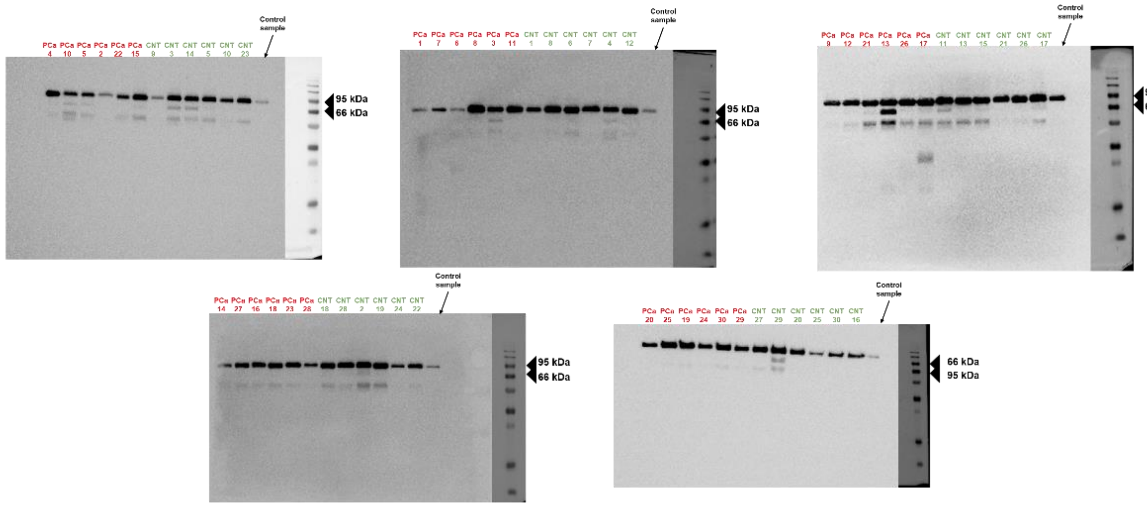

Transthyretin (TTR)

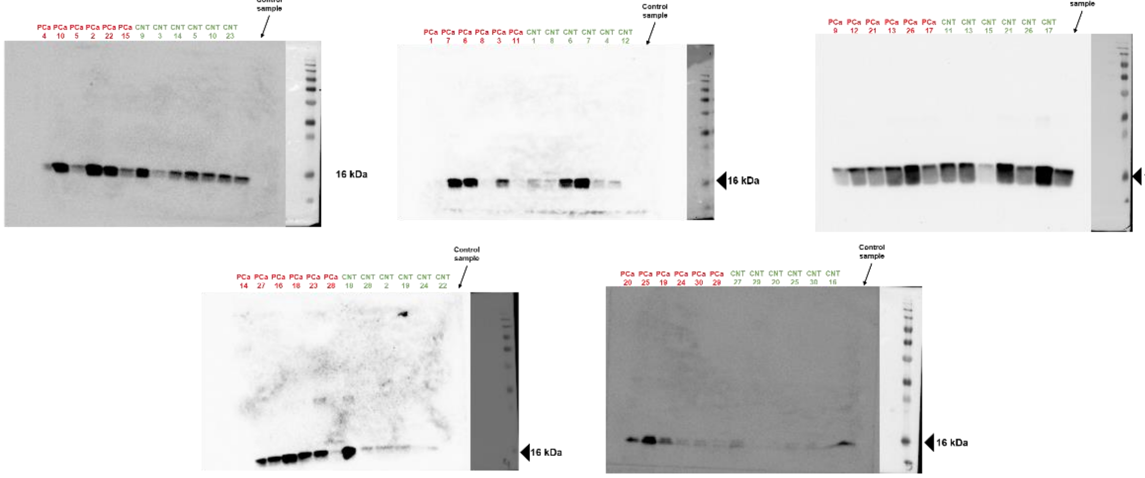

Figure S1. Original western blots figures.

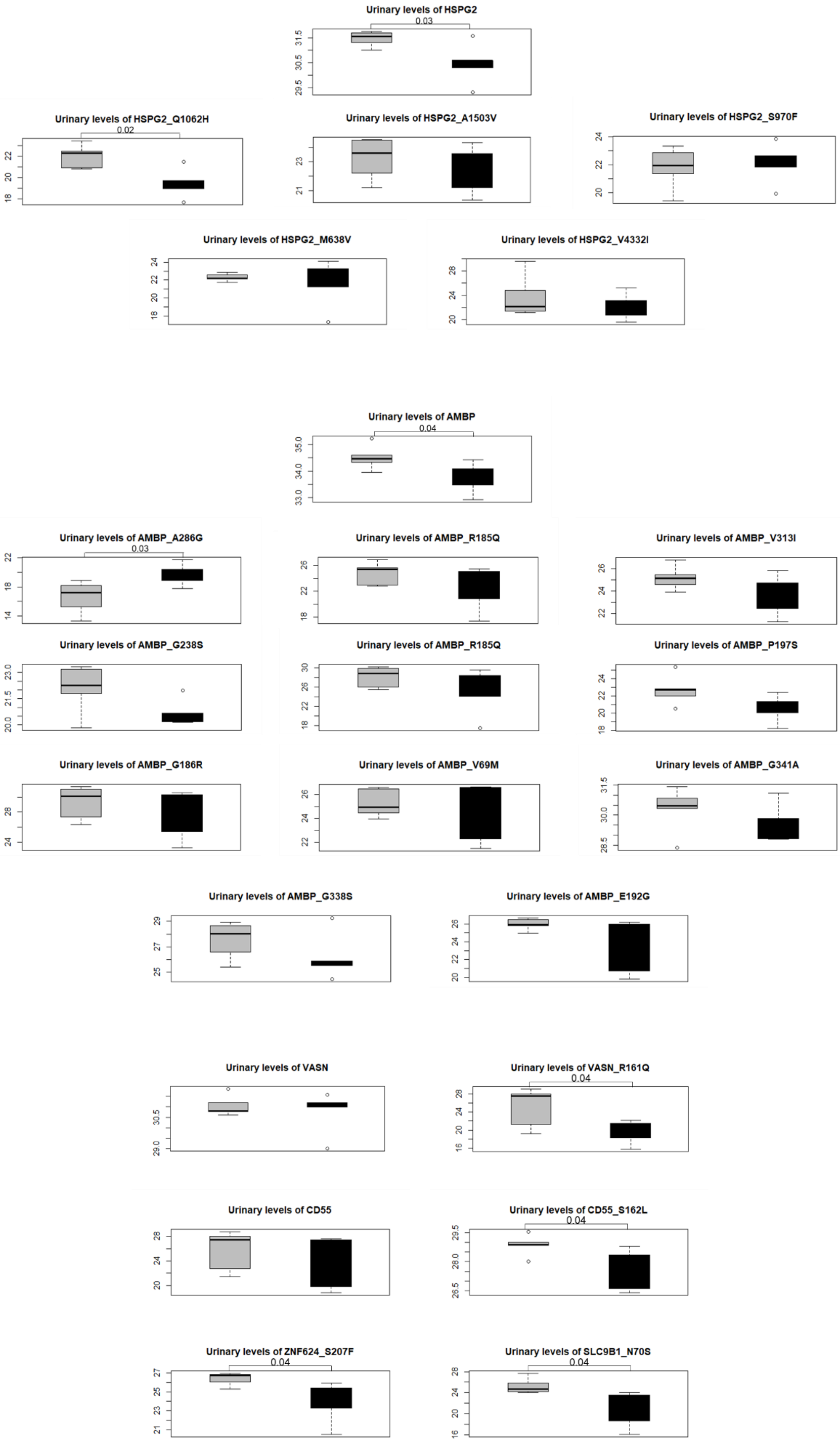

Figure S2. Levels of AMBP*A286G, SLC9B1*N70S, HSPG2*Q1062H, ZNF624*S207F, VASN*R161Q, and CD55* S162L mutant protein isoforms and respective levels of native form (when applicable) in the urine from PCa patients.
